# An Ala/Glu difference in E1 of Cx26 and Cx30 contributes to their differential anionic permeabilities

**DOI:** 10.1085/jgp.202413600

**Published:** 2024-09-20

**Authors:** Lina Kraujaliene, Tadas Kraujalis, Mindaugas Snipas, Vytas K. Verselis

**Affiliations:** 1 Institute of Cardiology, Lithuanian University of Health Sciences, Kaunas, Lithuania; 2 Department of Applied Informatics, Kaunas University of Technology, Kaunas, Lithuania; 3 Department of Mathematical Modelling, Kaunas University of Technology, Kaunas, Lithuania; 4 Dominick P. Purpura Department of Neuroscience, Albert Einstein College of Medicine, Bronx, NY, USA

## Abstract

Two closely related connexins, Cx26 and Cx30, share widespread expression in the cochlear cellular networks. Gap junction channels formed by these connexins have been shown to have different permeability profiles, with Cx30 showing a strongly reduced preference for anionic tracers. The pore-forming segment of the first extracellular loop, E1, identified by computational studies of the Cx26 crystal structure to form a parahelix and a narrowed region of the pore, differs at a single residue at position 49. Cx26 contains an Ala and Cx30, a charged Glu at this position, and cysteine scanning in hemichannels identified this position to be pore-lining. To assess whether the Ala/Glu difference affects permeability, we modeled and quantified Lucifer Yellow transfer between HeLa cell pairs expressing WT Cx26 and Cx30 and variants that reciprocally substituted Glu and Ala at position 49. Cx26(A49E) and Cx30(E49A) substitutions essentially reversed the Lucifer Yellow permeability profile when accounting for junctional conductance. Moreover, by using a calcein efflux assay in single cells, we observed a similar reduced anionic preference in undocked Cx30 hemichannels and a reversal with reciprocal Ala/Glu substitutions. Thus, our data indicate that Cx26 and Cx30 gap junction channels and undocked hemichannels retain similar permeability characteristics and that a single residue difference in their E1 domains can largely account for their differential permeabilities to anionic tracers. The higher anionic permeability of Cx26 compared with Cx30 suggests that these connexins may serve distinct signaling functions in the cochlea, perhaps reflected in the vastly higher prevalence of Cx26 mutations in human deafness.

## Teaser

Kraujaliene et al. used modeling and dye uptake/transfer studies to show that an Ala/Glu difference in the E1 domains of Cx26 and Cx30 can account for their differential permeabilities to anionic permeants. The permeability profiles are conserved in gap junction channels and hemichannel configurations.

## Introduction

Connexins (Cxs) are a family of homologous integral membrane proteins that comprise the subunits of intercellular gap junction (GJ) channels. Six Cx subunits assemble to form a hemichannel (or connexon) and two hemichannels from apposing cells dock to form an intercellular GJ channel. Aside from the transmission of electrical signals, GJ channels take on a broader role in intercellular chemical communication due to a typically large pore size that can accommodate a host of signaling molecules. These roles also extend to hemichannels that can function in the absence of docking, thereby mediating electrical and chemical signaling across the plasma membrane.

The cochlea expresses two Cxs, Cx26 and Cx30, throughout the various supporting cells of the Organ of Corti as well as the cells that make up the lateral wall (Ahmad et al., 2003; Forge et al., 2003a; Lautermann et al., 1998; Liu et al., 2017; Liu and Zhao, 2008; Zhao and Yu, 2006). Mutations in either of these Cxs have been shown to be causally linked to congenital, sensorineural hearing loss, and a heterogeneous array of skin disorders (Delmar et al., 2018; Laird and Lampe, 2022; Srinivas et al., 2018; Wingard and Zhao, 2015). For hearing loss, the vast majority of mutations occur in Cx26, and its importance in auditory function is evidenced by the fact that Cx26 mutations account for ∼50% of severe-to-profound inherited deafness cases across diverse ethnic populations (Angeli et al., 2012; Apps et al., 2007; Chan and Chang, 2014; Duman and Tekin, 2012). Animal studies have affirmed the critical nature of Cx26 in hearing, demonstrating that genetic deletion of Cx26 in mice invariably results in profound deafness whereas deletion of Cx30 does not if high enough levels of Cx26 are maintained (Ahmad et al., 2007; Boulay et al., 2013; Qu et al., 2012). Thus, although Cx26 can replace Cx30 to preserve hearing, Cx30 cannot replace Cx26. These data suggest that functional differences between Cx26 and Cx30 channels may contribute to their differential impacts on hearing loss.

A reported functional distinction between Cx26 and Cx30 GJs relates to their differing permeability characteristics determined from tracer flux studies showing that Cx26 GJs, but not Cx30 GJs, are permissive to larger anionic molecules (Beltramello et al., 2003; Jagger and Forge, 2006; Manthey et al., 2001; Sun et al., 2005; Yum et al., 2007; Zhao, 2005). In a companion paper to this study, another distinction relates to their functions as undocked hemichannels (Sanchez et al., 2024). Over a broad range of extracellular Ca^2+^ concentrations, Cx30 functions relatively poorly as an undocked hemichannel compared with Cx26. Poor Cx30 hemichannel function was attributed to interactions between adjacent charged residues, Glu49 and Asp50, that bias hemichannels toward closure. This charge pair is absent in Cx26, which contains an Ala at position 49, and substitutions neutralizing either residue in Cx26 or Cx30 resulted in robust hemichannel function. These two residues are positioned in the proximal segment of the first extracellular loop, E1, a region that forms a substantive segment of Cx channel pores and participates in gating (Bargiello et al., 2018; Kwon et al., 2012; Tang et al., 2009; Verselis et al., 2009). Using the substituted-cysteine-accessibility-method, position 49 was shown to be pore-lining in biologically active Cx26 and Cx30 hemichannels (Sanchez et al., 2024). Moreover, Cys- substitution at position 49 led to disulfide formation and high-affinity metal binding in hemichannels, indicative that this residue is situated in a narrowed, flexible region of the pore.

Given the high potential for position 49 to influence ionic flux through the pore, we examined the effect of the Ala/Glu difference in Cx26 and Cx30 GJ channels on their differential permeabilities to anionic tracers. Indeed, we found that switching the Ala/Glu residues in Cx26 and Cx30 largely switched the permeability profiles of the respective GJs. This finding extended to undocked hemichannels, indicating a conserved role for the Ala/Glu difference in governing permeability in both GJ channel and hemichannel configurations. The combined effects of the Ala/Glu difference on the gating and permeability characteristics Cx26 and Cx30 suggest a potentially key role in conferring different signaling functions to these Cxs in the cochlea.

## Materials and methods

### Construction of Cx26 and Cx30 mutants

Human wild-type (WT) Cx26 and Cx30 were cloned into the BamHI restriction site of the pCS2^+^ expression vector for functional studies and exogenous expression. To attach the monomeric fluorescent proteins msfGFP and mScarlet to the carboxy termini of Cx26 and Cx30 respectively, the nucleotide sequence of msfGFP or mScarlet was fused in frame via a seven amino acid linker (5′-ACG​CGT​ACG​CGG​CCG​CTC​GAG-3′). Cx-fusion proteins and site-directed mutations were constructed by GenScript. All constructs were verified by sequencing.

### Exogenous expression of connexins

For Cx expression in mammalian cell lines, we used HeLa cells (CCL-2; ATCC). WT Cx26 and Cx26(A49E) were tagged with msfGFP, and WT Cx30 and Cx30(E49A) were tagged with mScarlet. Cells were grown in Dulbecco’s modified Eagle media (DMEM; Sigma-Aldrich) supplemented by 10% of fetal bovine serum (Sigma-Aldrich) and 1% of penicillin, 10,000 U and 10 mg streptomycin/ml solution (Sigma-Aldrich). Lipofectamine 2000 (Invitrogen) and/or jetPrime (Polyplus transfection) reagents were used for transient transfections, which were performed on the second day after seeding cells on coverslips in Petri dishes. Experiments were carried out 48 h after transfection.

### Electrophysiological recordings

For electrophysiological recordings, HeLa cells were grown on glass coverslips and transferred to an experimental chamber placed on the stage of an Olympus IX70 inverted microscope (Olympus) equipped with a fluorescence imaging system and a constant flow-through perfusion. Single and dual whole-cell patch-clamp methods were used to record currents from hemichannels and GJ channels, respectively, using EPC-7 (HEKA) or EPC-8 (HEKA) patch-clamp amplifiers. Hemichannel recordings were obtained from voltage steps or ramps applied to single, isolated cells clamped in a whole-cell configuration. GJ channel recordings were obtained from cell pairs that showed fluorescent plaques in areas of cell contact. For measurements of GJ conductance (g_j_), each cell in a pair was clamped independently in a whole-cell patch configuration to a common holding potential. Transjunctional voltages, V_j_s, were applied by stepping the voltage in one cell while keeping the voltage in the other cell constant. Junctional current (I_j_) was measured as the current change in the unstepped cell. To obtain g-V_m_ relationships of hemichannels, voltage ramps were applied over a wide voltage range.

Patch pipettes were pulled from borosilicate glass capillary tubes with filaments using P-97 micropipette puller (Sutter Instrument Co.). Pipette resistance was maintained between 2 and 3 MΩ to minimize the effects of series resistance on current measurements. Extracellular solutions consisted of modified Krebs-Ringer solutions, one containing divalent cations (MKRS) and another in which no divalent cations were added (divalent-free solution, DCFS). MKRS contained (in mM): 140 NaCl, 4 KCl, 2 CaCl_2_, 1 MgCl_2_, 2 CsCl, 1 BaCl_2_, 5 HEPES, 5 glucose, and 2 pyruvate, pH 7.8. DCFS had the same composition but excluded CaCl_2_ and MgCl_2_. Patch pipettes were filled with a solution containing (in mM): 130 CsCl, 10 NaAsp, 0.26 CaCl_2_, 5 HEPES, 2 BAPTA, and 1 MgCl_2_, pH 7.7.

Data were acquired with AT-MIO-16X D/A boards from National Instruments using custom acquisition and analysis software (VTDaq, NexusWiz, written by E. Brady Trexler, Gotham Scientific, Hasbrouck Heights, NJ, USA).

### Calcein efflux assay

Calcein-AM (Millipore Sigma) was prepared as a 5 mM stock solution in DMSO. Cells were incubated with the stock solution diluted to 25 µM in MKRS for 30 min at room temperature, then washed twice with MKRS. For WT Cx26 and Cx26-A49E tagged with msfGFP, brightfield and fluorescence images were taken of a field of cells prior to incubation with calcein to provide clearer identification of transfected and non-transfected cells as calcein excitation and emission wavelengths overlap with msfGFP. To monitor calcein efflux, changes in intracellular fluorescence intensities were recorded every 10 s (100 ms exposure time) for the duration of the recordings (typically ∼30 min). Experiments were performed on an inverted Olympus IX70 (Olympus) microscope using an ORCA digital camera (Hamamatsu Photonics) and UltraVIEW (PerkinElmer Life Sciences) imaging software. Time-lapse images were taken using an Olympus 60× PlanApo, 1.40 N.A. Oil objective. The excitation filter of 482/35 nm and emission filter of 535/45 nm wavelength were used for measurements of calcein fluorescence and for visualization of Cx with msfGFP tag. An excitation filter of 605/55 nm and an emission filter of 540/25 nm wavelength were used for visualization of Cx with mScarlet tag. All images were acquired at room temperature in MKRS. To obtain better contrast, the grey levels were adjusted in UltraVIEW imaging software before merging images with different colors and exporting them to a 8-bit format.

### Lucifer Yellow permeability measurements through GJs

Lucifer Yellow (LY) CH dipotassium salt (Millipore Sigma) was dissolved in the pipette solution to a 2 mM working concentration. LY was introduced into one cell of a pair (source cell) by establishing a whole-cell patch clamp recording using a pipette filled with the LY solution. Fluorescent intensity was registered in both source and recipient cells using time-lapse imaging. Recording duration was set to 15 min, with fluorescence recorded every 30 s using 100 ms exposure times to minimize bleaching. At the end of the time-lapse recording, a whole-cell patch was established in the recipient cell to evaluate junctional conductance (g_j_). Experiments were performed using the same imaging hardware and software as indicated for calcein measurements with an excitation filter of 425/20 nm and an emission filter of 540/25 nm wavelength for LY.

### General mathematical model for fluorescent dye flux through GJ channels and hemichannels

The flux of a fluorescent dye between two compartments (in this case between an isolated cell and the bathing medium or between two coupled cells) was modeled according to Fick’s law in which flux is proportional to the concentration gradient between the two compartments. Thus, flux can be described by the following system of ordinary differential equations (ODEs):

$$\left\{ \begin{array}{l} \;\frac{dC_{1}\left(t\right)}{dt}=\frac{P∙\left[C_{2}\left(t\right)-C_{1}\left(t\right)\right]}{Vol_{1}}\\ \;\frac{dC_{2}\left(t\right)}{dt}=\frac{P∙\left[C_{1}\left(t\right)-C_{2}\left(t\right)\right]}{Vol_{2}} \end{array}\right.$$ (1)

Here, C_1_(t) and C_2_(t) denote the concentrations of the two compartments. The constant *P* is the total permeability across the cell membrane. *Vol*_*1*_ and *Vol*_*2*_ denote the volumes of the two compartments.

In the experimentally used concentration range of the dyes we used, LY and calcein, fluorescence intensity was shown to depend linearly on concentration; calcein self-quenching only occurs at concentrations exceeding 4 mM (Hamann et al., 2002; Trexler et al., 2000). Thus, *C(t)* linearly follows the changes in fluorescence intensity, *F*(*t*), and can be described by the following relationship:

$$F\left(t\right)=f∙C\left(t\right)+f_{0}$$ (2)

Here, *f* is a constant that denotes fluorescence per unit concentration and *f*_0_ is the background fluorescence, which was measured in each of the recordings in regions devoid of cells.

### Modeling calcein efflux through Cx hemichannels

To model the efflux of calcein through Cx hemichannels, the system of ODEs in Eq. 1 can be simplified because the calcein concentration in the bathing medium can be considered negligible due to this compartment acting as an infinite volume. Designating *C*_*1*_ as *C*_*in*_, *C*_*2*_ as *C*_*out*_ = 0 and *Vol*_*1*_ as *Vol*_*in*_, the changes in intracellular calcein concentration, *C*_*in*_(*t*), can be described by the following ODE:

$$\frac{dC_{in}\left(t\right)}{dt}=\frac{-P∙C_{in}\left(t\right)}{Vol_{in}}$$ (3)

Here, *P* is the total permeability, which is also the product of single hemichannel permeability, *P*_*γ*_, the number of hemichannels, *n*, and hemichannel open probability, *P*_*o*_. Likewise, macroscopic hemichannel conductance, *g*, is the product of single hemichannel conductance, γ, the number of hemichannels, *n*, and hemichannel open probability, *P*_*o*_. Thus:

$$P=P_{\gamma }∙n∙P_{o}=P_{\gamma }∙\frac{g}{\gamma }$$ (4)

which means that *P*_*γ*_ can be evaluated from *P/(g/γ*). Following loading of cells and washout of calcein-AM from the bath, a slight increase in *F*_*in*_*(t)* could be observed in some instances, which could be due to continued esterase cleavage of residual calcein-AM. To account for this increase, we included an additional positive term *α* into the mathematical model:

$$\frac{dC_{in}\left(t\right)}{dt}=\frac{\alpha -P∙C_{in}\left(t\right)}{Vol_{in}}$$ (5)

The modified ODE in Eq. 5 has the following analytical solution:

$$C_{in}\left(t\right)=\frac{\alpha }{P}-\left(\frac{\alpha }{P}-C_{in}\left(0\right)\right)∙e^{-\frac{P}{Vol_{in}}∙t}$$ (6)

To obtain model parameters, fits were obtained during the phase of the recording that exhibited a linear change in fluorescence, *F*_*in*_*(t)*, which could be adequately reproduced by approximating Eq. 6 using the Taylor series expansion of the exponential term and gives the following solution:

$$F_{in}\left(t\right)=F_{in}\left(t_{0}\right)+f∙\left(\frac{\alpha -C_{in}\left(t_{0}\right)∙P}{Vol_{in}}\right)∙\left(t-t_{0}\right)$$ (7)

Thus, starting at time *t*_*0*_, *F*_*in*_*(t)* can be fit by a linear function *F*_*in*_(*t*) = *k t* + *c*. The slope *k* reflects the quantity *f*∙(*α-C*_*in*_*(t*_*0*_*)∙P)/Vol*_*in*_. Because *α*, *Vol*_*in*_, and *f* (the fluorescence per unit of concentration) are Cx-independent, higher negative values of the fitted slope *k* correlate with higher total permeability, and thus with single hemichannel permeability, *P*_*γ*_, when *g/γ* is evaluated to provide *n*∙*P*_o_.

### Modeling intercellular flux of LY through GJ channels

Cell-1 (source cell) and the patch pipette containing LY can be considered as a single well-mixed compartment after reaching equilibration. Thus, following an initial rise in concentration and equilibration with the pipette, cell-1 can be considered to exhibit a constant concentration, *C*_*1*_*(t)* = *C*_*1*_. Thus, the kinetics of the concentration changes of LY in the recipient cell, *C*_2_(t) can be described by the following ODE:

$$\frac{dC_{2}\left(t\right)}{dt}=\frac{P_{j}∙\left(C_{1}-C_{2}\left(t\right)\right)}{Vol_{2}}$$ (8)

where *P*_*j*_ is total junctional permeability. This ODE has the following analytical solution:

$$C_{2}\left(t\right)=C_{1}-\left(C_{1}-C_{2}\left(t_{0}\right)\right)∙e^{-\frac{P_{j}}{Vol_{2}}∙\left(t-t_{0}\right)}$$ (9)

Thus, the relative increase in fluorescence intensity in cell-2 is given by:

$$\frac{F_{2}\left(t\right)}{F_{2}\left(t_{0}\right)}=\frac{C_{1}}{C_{2}\left(t_{0}\right)}-\left(\frac{C_{1}}{C_{2}\left(t_{0}\right)}-1\right)∙e^{-\frac{P_{j}}{Vol_{2}}∙\left(t-t_{0}\right)}$$ (10)

**Figure S1.**
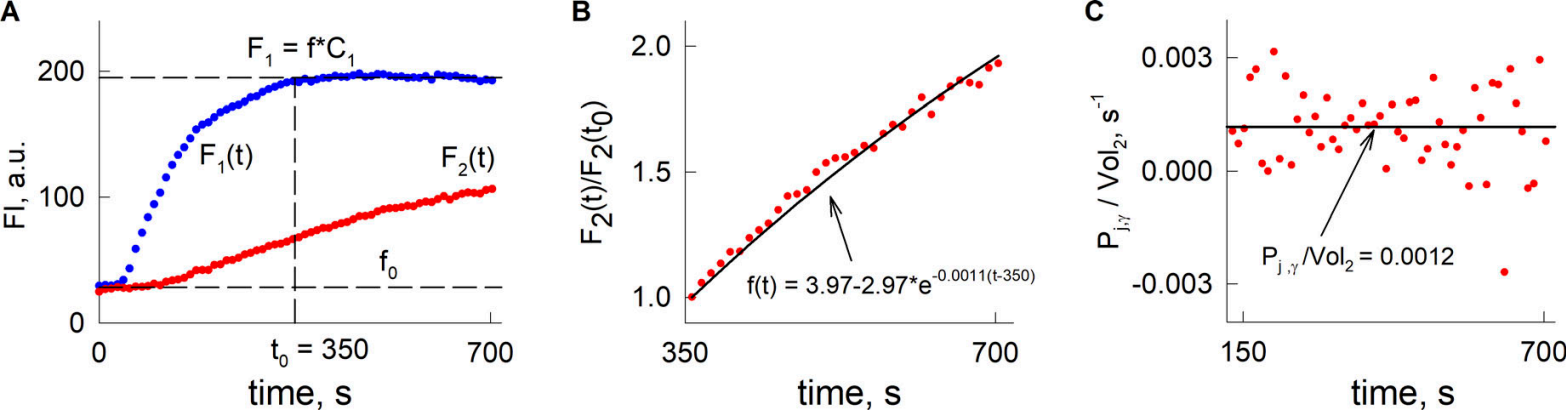
**Model-based evaluation of LY permeation through GJ channels. (A)** A representative example of changes in LY fluorescence intensity in an electrically coupled cell pair. The blue trace, *F*_*1*_*(t)*, represents the fluorescence in the cell patched with the electrode containing LY. In this cell pair, *F*_*1*_*(t)* reached a steady-state level after ∼350 s, which indicates that the concentration of LY in the patch pipette and the cell reached equilibrium. **(B)** The red circles represent changes in LY fluorescent intensity in the unpatched cell, *F*_*2*_*(t)*, taken at time points after which a steady-state level was reached in the patched cell. The solid line represents a fit of the data to the mathematical model (see Materials and methods, Eq. 10). The inverse of the time constant of the fitted exponential, 0.0011, provides an estimate for *P*_*j,*_*/Vol*_*2*_. **(C)** This shows an illustration of an alternative method for the estimation of *P*_*j*_*/Vol*_*2*_, based on the discretization of the ODE, which describes the flow of permeable dyes through a GJ (see Materials and methods, Eq. 11).

Fitting this theoretical curve to the experimental data (see example in Fig. S1) gives an estimate of a single parameter, the ratio of the total permeability *P*_*j*_, and the volume of the recipient cell, *Vol*_*2*_. In some of the dye transfer experiments, fluorescence in the donor cell, *F*_*1*_*(t)*, did not reach a plateau steady-state value. In these cases, we used an approximate estimate of *P*_*j*_*/Vol*_*2*_ based on the following discretization of the ODE from Eq. 8:

$$\frac{P_{j}}{Vol_{2}}\approx \frac{F_{2}\left(t_{i}\right)-F_{2}\left(t_{i-1}\right)}{\left(\frac{F_{1}\left(t_{i}\right)-F_{2}\left(t_{i}\right)+F_{1}\left(t_{i-1}\right)-F_{2}\left(t_{i-1}\right)}{2}\right)∙\left(t_{i}-t_{i-1}\right)}$$ (11)

This method was applied in the previous studies of GJ channel permeability (Kanaporis et al., 2011). Using this approximate formula, estimates of *P*_*j*_*/Vol*_*2*_ depend on the selected time point *t*_*i*_. Thus, we took an average value of all the estimates obtained after *t*_*i*_ reached the ∼2 min mark, when there was no longer a large variation in the estimates (see representative example in Fig. S1 C).

*P*_*j*_, which reflects the total GJ permeability between a cell pair, can be expressed similarly as in Eq. 4 to give single GJ channel permeability by assessing g_j_/γ_j_, total GJ conductance divided by the single channel conductance, giving the parameter for the permeability of a single GJ channel (*P*_*j,γ*_*/Vol*_*2*_).

### Statistical analysis

For group comparisons, we applied either one-way ANOVA with post hoc Tukey’s test or, if a test of normality failed, the Kruskall–Wallis test (i.e., ANOVA on ranks) with post hoc Dunn’s test. Pairwise comparisons were performed using the Mann–Whitney rank sum test. To test for normality, we used Shapiro–Wilk’s test. Statistical analysis was performed using R version 4.3.3 via RStudio. The detailed results of the performed statistical analysis are presented in Tables S1 and S3.

### Online supplemental material

**Figure S2.**
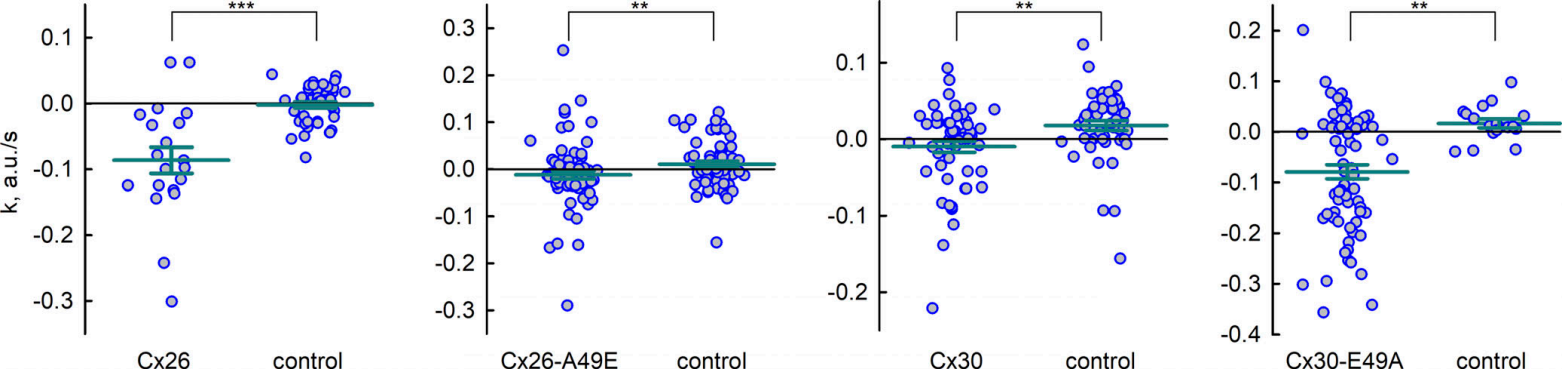
**Statistical comparison of calcein efflux in Cx-expressing versus control cells.** Summary of data comparing calcein efflux in cells exhibiting Cx-expression and those in which Cx expression was below levels of detection (designated as controls). Bar graphs show the model parameter *k*, the slope of the change in fluorescence, for each hemichannel type in Cx-expressing and control cells from the same dishes. All cells with visible Cx expression showed evidence of increased calcein efflux compared to control cells P values of Mann–Whitney rank sum test: Cx26–2.75e−05, Cx26(A49E)–0.005, Cx30–0.002, Cx36(E49A)–0.002. Error bars represent means and standard errors, and circles represent individual data points; the number of recordings was 20 for Cx26 and 44 for control, 74 for Cx26(A49E) and 63 for control, 57 for Cx30 and 50 for control, 72 for Cx30(E49A) and 17 for control. Asterisks denote statistical significance (**P value <0.01; ***P value <0.001).

Fig. S1 shows illustrations and representative examples of model-based evaluation of data obtained from the dye flux assay through GJs. Fig. S2 summarizes data comparing calcein efflux in Cx-expressing and non-expressing cells. Table S1 presents data of statistical analyses for pairwise comparison of Lucifer Yellow permeability coefficients. Table S2 summarizes the results of statistical analyses for pairwise comparison of the estimated number of functional hemichannels. Table S3 shows the results of statistical analyses for pairwise comparison of calcein permeability coefficients.

## Results

### The differential anionic permeability profile of Cx26 and Cx30 GJ channels is reversed by swapping the Ala/Glu residues at position 49

**Figure 1.**
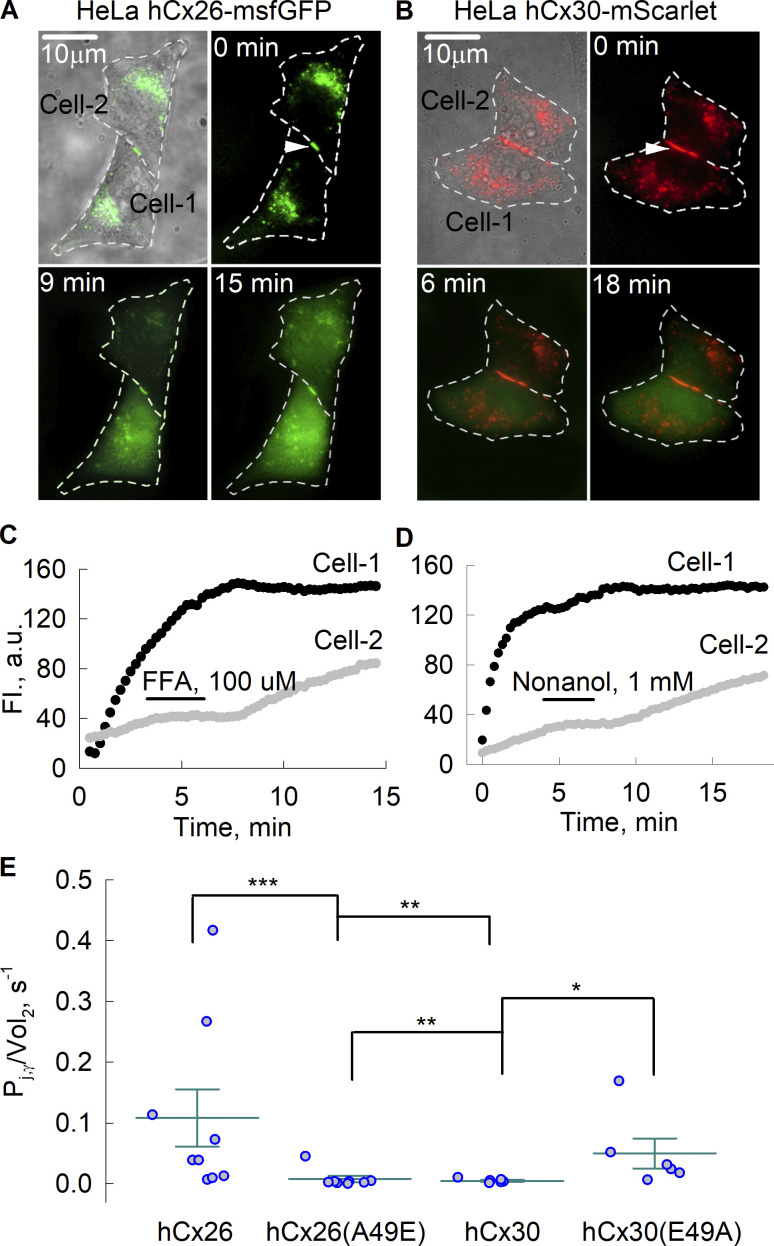
**The transfer of lucifer yellow (LY) through GJ channels formed of Cx26, Cx30, and their Ala/Glu variants. (A and B)** Images of Cx-expressing HeLa cell pairs were obtained at different times during LY transfer experiments. Cell boundaries are indicated by the dotted outlines. The fluorescence of the msfGFP tag on Cx26 is shown in green (A) and the mScarlet tag on Cx30 is shown in red (B). LY fluorescence is also shown in green. White arrows indicate GJ plaques. Scale bars indicate 10 µm. **(C and D)** Representative examples of plots of changes in LY fluorescence over time in Cx26-msfGFP and Cx30-mScarlet cell pairs. The flux of LY from the donor cell (Cell-1) was evident by the increase in fluorescence over time in the recipient cell (Cell-2). This increase was interrupted upon the application of GJ channel inhibitors flufenamic acid (FFA) and nonanol (indicated by the bars). The rise in fluorescence resumed after washout. **(E)** Statistical comparison of the estimates of the ratio of a single GJ channel permeability to the cell volume, *P*_*j,γ*_*/Vol*_*2*_, using Kruskal–Wallis test with post hoc Dunn’s test. The error bars represent means and standard errors and circles represent individual data points; the values of *P*_*j,γ*_*/Vol*_*2*_ were 0.109 ± 0.05 (*n* = 9) for Cx26, 0.008 ± 0.05e−4 for Cx26(A49E) (*n* = 8), 0.005 ± 0.01e−4 for Cx30 (*n* = 6), and 0.05 ± 0.03e−3 for Cx30(E49A) (*n* = 6). Asterisks denote statistical significance (**P value <0.01; ***P value <0.001).

We quantified LY permeability in HeLa cells by measuring the transjunctional flux of LY and junctional conductance, g_j_, in the same cell pairs and fitting the data to a two-compartment model (see Materials and methods). We utilized tagged versions of Cxs, Cx26-msfGFP, and Cx30-mScarlet to enable the selection of cell pairs containing GJ plaques. The cell pairs chosen typically exhibited a modest-sized GJ plaque at the appositional membrane as illustrated in Fig. 1, A and B (arrows). Dye fluorescence in the donor (Cell-1) and recipient (Cell-2) cells is shown at three time points starting at *t* = 0, which is when a whole-cell recording was established in the donor cell. Fig. 1, C and D show examples of plots of fluorescence over time in donor and recipient cells. Following the establishment of a whole-cell recording in the donor cell, fluorescence rose rapidly and typically plateaued in several minutes. Transfer to the recipient cell was evident for both Cx26-msfGFP and Cx30-mScarlet cell pairs. Application and washout of GJ blockers flufenamic acid or nonanol temporarily interrupted the dye flow indicating that transfer was mediated by GJs rather than cytoplasmic bridges. Measurement of g_j_ at the end of the experiment and fits of the data to the model (see Materials and methods) yielded values for permeability expressed as *P*_*j,γ*_*/Vol*_*2*_ (Fig. 1 E). Although we saw evidence of LY transfer for both Cx26 and Cx30 when quantified using our model, permeability to LY was found to be considerably higher for Cx26-msfGFP than for Cx30-mScarlet, consistent with the published studies as previously indicated. Of note, tagging the C-terminal domain of Cx26 and Cx30 was shown to have no effect on GJ channel permeability (Beltramello et al., 2005). When we quantified LY transfer for the variants that swapped the Ala/Glu residues at position 49, the permeability profiles for LY reversed. Cx26(A49E)-msfGFP GJs now poorly transferred LY, much like Cx30-mScarlet GJs, and conversely, Cx30(E49A)-mScarlet GJs now showed substantial LY transfer. Thus, the amino acid difference at position 49 between Cx26 and Cx30 plays an important role in their differential LY permeability profiles.

### Cx26 and Cx30 hemichannels also show an anionic preference that is reversed by swapping the Ala/Glu residues at position 49

**Figure 2.**
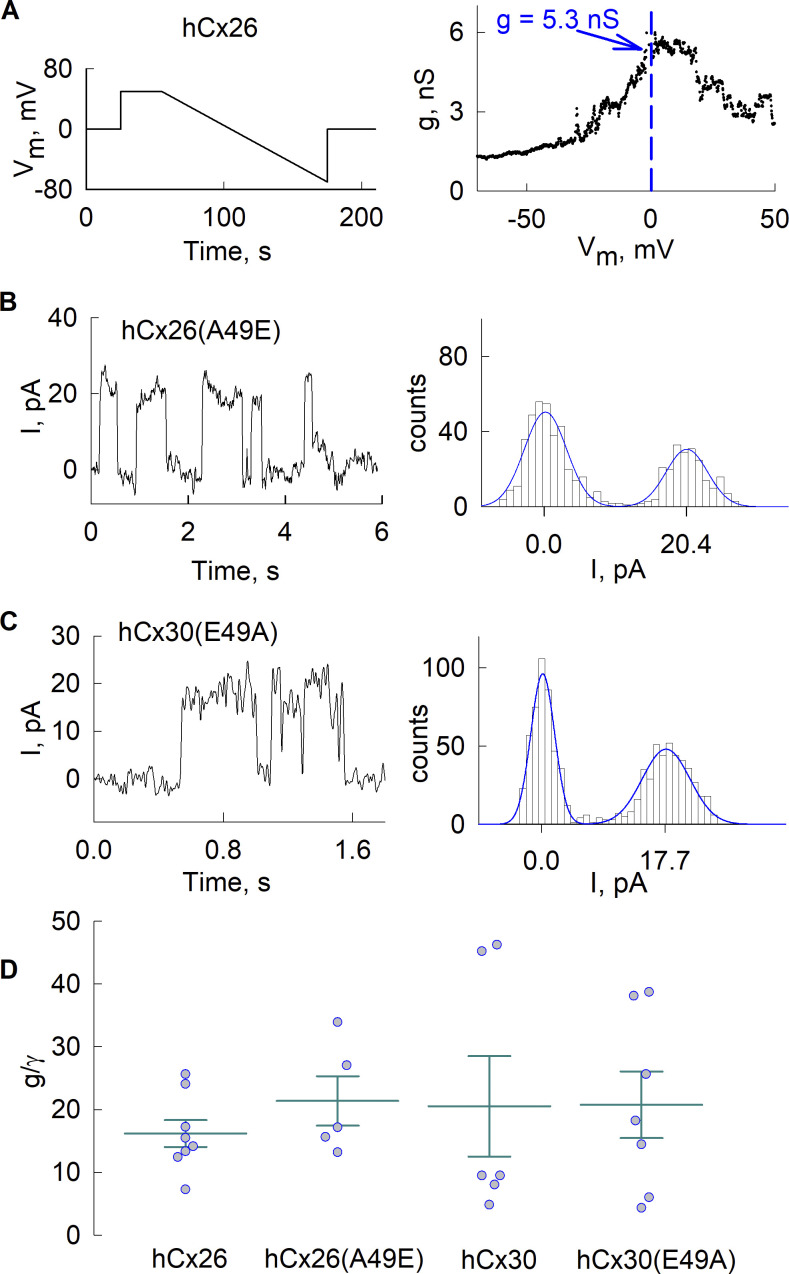
**Evaluation of the number of functioning hemichannels in HeLa cells. (A)** Representative example of an electrophysiological whole-cell patch recording in a HeLa cell expressing Cx26 used to evaluate hemichannel conductance, g, at V_m_ = 0 mV (dashed vertical line). The left panel shows the applied voltage protocol and the right panel shows the resulting g-V_m_ relationship. **(B and C)** Electrophysiological recordings (left panels) in low-expressing cells show visible unitary events in HeLa cells expressing Cx26(A49E) (B) and Cx30(E49A) (C). Currents were leak-subtracted from the mean current levels assessed from the closed events. Corresponding all-point amplitude histograms are shown on the right of each recording. Solid blue lines show appropriately scaled fits of mixtures of normal distributions. Mean unitary conductance assessed was 340 ± 11 pS (*n* = 5) and ∼295 ± 9 pS (*n* = 5) Cx26(A49E). **(D)** Comparison of the number of functioning hemichannels estimated as a ratio of overall hemichannel conductance and unitary conductance, g/γ. Error bars represent means and standard errors, and circles represent individual data points; number of recordings was 8 for Cx26, 6 for Cx26(A49E), 6 for Cx30, and 7 for Cx30(E49A). One-way ANOVA with post hoc Tukey’s test did not show significant differences among Cxs (P value = 0.863).

Next, we wanted to assess whether the permeabilities of undocked hemichannels behaved in a similar manner as the corresponding GJ channels. For dye flux studies in hemichannels, we utilized a negatively charged dye, calcein, which is available as a membrane-permeant acetoxymethyl ester, calcein-AM, thereby allowing loading of cells without patching and the assessment of efflux in many cells following application of divalent cation-free solution (DCFS). To quantify single hemichannel permeability, *P*_*γ*_, we performed a separate set of electrophysiological measurements to estimate the number of open hemichannels. g-V_m_ relationships were obtained by applying voltage ramps from +50 to −70 mV, 2 min in duration, in DCFS. Conductance was evaluated at V_m_ = 0 mV for cells expressing Cx26-msfGFP, Cx30-mScarlet, and the reciprocal Ala/Glu variants (see a representative example in Fig. 2 A for a cell expressing Cx26-msfGFP). These measurements provided mean values for hemichannel conductance under the same conditions as in the calcein efflux experiments. We also assessed the unitary conductances of the Ala/Glu variants from whole-cell recordings in cells expressing low currents and visible unitary events. The mean value for Cx26(A49E)-msfGFP was ∼340 ± 11 pS, and for Cx30(E49A)-mScarlet was ∼295 ± 9 pS (Fig. 2, B and C), which were provided by fits of mixtures of normal distributions to all-point amplitude histograms. These values correspond well to published results for WT Cx26 (340 pS) and Cx30 (283 pS) hemichannels measured as slope conductances at V_m_ = 0 (Sánchez et al., 2010; Valiunas and Weingart, 2000). Thus, unitary conductance did not change appreciably when the Ala/Glu residues at position 49 were reciprocally exchanged between Cx26 and Cx30. Fig. 2 D shows mean values for macroscopic conductance divided by the value for unitary conductance, *g/γ*, for each hemichannel type, which provides a measure of the number of hemichannels multiplied by hemichannel open probability, *n∙P*_*o*_. Statistical analyses did not show significant differences among the group medians for each of the WT and variant hemichannels (P val. = 0.788), and no significant differences in pairwise comparisons of sample distributions (the lowest P val. of 0.099 were obtained for a pairwise comparison of Cx26(A49E) and Cx30 hemichannels). Hence, the differences in the estimated efflux rates will largely reflect differences in the permeability properties of these Cx hemichannels.

**Figure 3.**
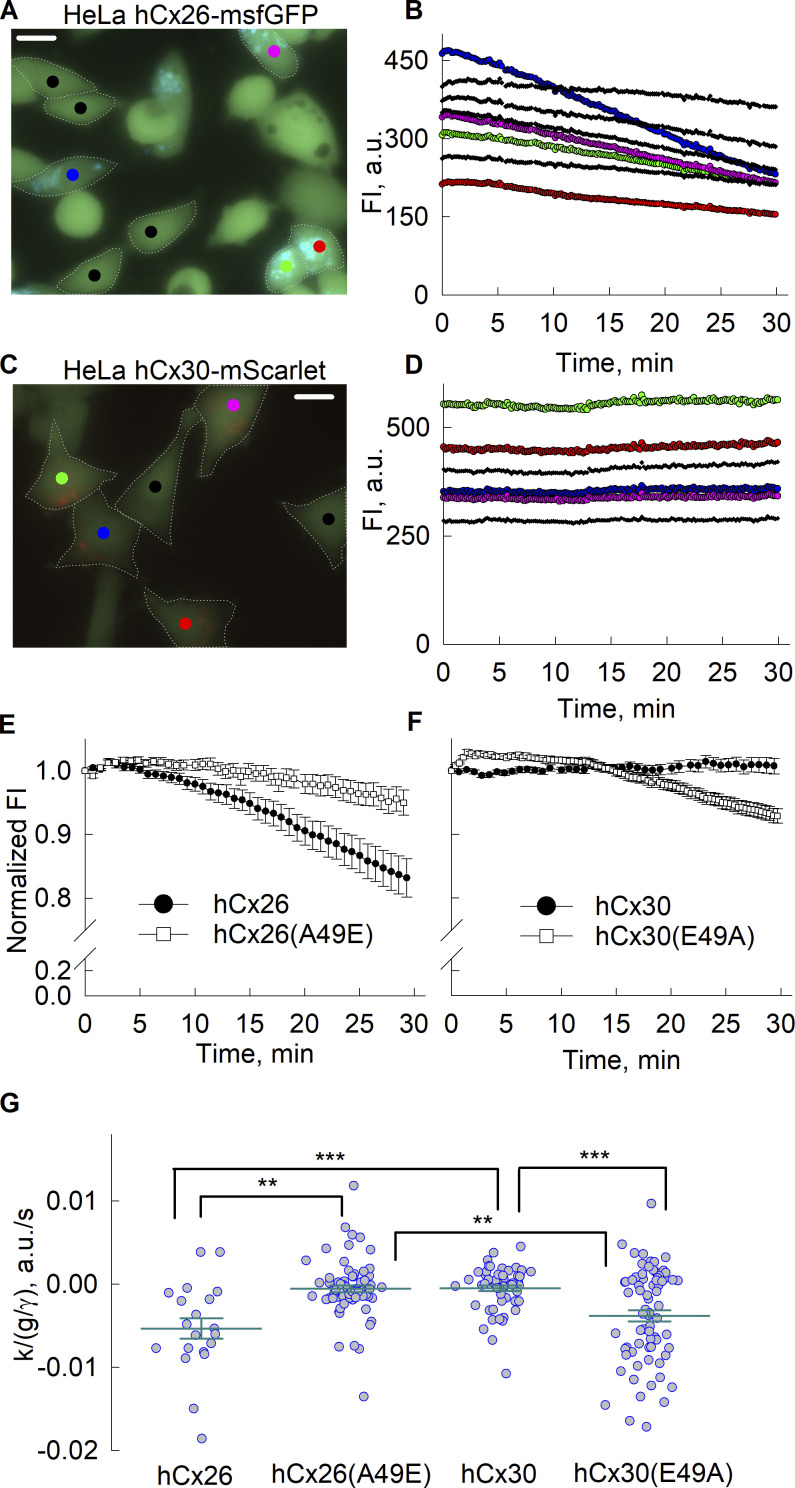
**The measurements of calcein efflux through hemichannels. (A)** An image of HeLa cells loaded with calcein (green). Cells expressing Cx26-msfGFP show punctate fluorescence (depicted in blue). Regions of interest (ROIs) are shown as filled circles of different colors placed on individual cells. Scale bar indicates 10 µm. **(B)** Plots fluorescence intensity over time obtained from each of the indicated ROIs; colors correspond to those of the ROIs with black representing cells lacking obvious evidence of Cx26-msfGFP expression. Cells were exposed to a solution free of added divalent cations at the beginning of the recording. **(C and D)** Similar to A, but for cells expressing Cx30-mScarlet (red signal). Scale bar indicates 10 µm. **(E)** Comparison of calcein efflux in cells expressing Cx26-msfGFP versus Cx26(A49E). Data represents changes in normalized fluorescence over time. **(F)** Same as in E but comparing calcein efflux in cells expressing Cx30-mScarlet versus Cx30(E49A)-mScarlet. **(G)** Summary of data obtained from fits of calcein efflux to the mathematical model. The model parameter *k*, the slope of the decline in fluorescence, divided by the average number of open hemichannels, *g/γ*, provided a measure of the permeability of a single hemichannel to calcein; a more negative value indicates higher permeability to calcein. Group comparison was performed using Kruskal–Wallis test with post hoc Dunn’s test. Error bars represent means and standard errors, and circles represent individual datapoints; the number of recordings was 20 for Cx26, 74 for Cx26(A49E), and 57 for Cx30, 72 for Cx30(E49A). Asterisks denote statistical significance (*P value <0.1, **P value <0.01; ***P value <0.001).

Quantitative results of the calcein efflux assay are shown in Fig. 3. Representative images of cells loaded with calcein are shown for WT Cx26-msfGFP and Cx30-mScarlet along with changes in calcein fluorescence, FI, over time measured from regions of interest placed within cells expressing Cx (colored) and those showing little or no-evidence of Cx expression (black); the latter are considered as controls. For Cx26-msfGFP (Fig. 3, A and B), the decrease in FI over time was notable and was considerably larger in cells expressing Cx26-msfGFP (colored versus black traces). In contrast, FI remained fairly constant in cells expressing Cx30-mScarlet, similar to control cells (Fig. 3, C and D). A statistical comparison of calcein efflux in Cx-expressing and control cells is provided in the supplement (Fig. S2).

Superimposition of the averaged normalized traces of FI over time shows a direct comparison of the effects of substitution at position 49 on Cx26 and Cx30 hemichannel permeability (Fig. 3, E and F). An A49E substitution in Cx26 substantially reduced calcein efflux compared to Cx26 whereas the E49A substitution in Cx30 now showed substantial calcein efflux not evident for Cx30. The results were quantified from fits to the parameter *k*, from *F*_*in*_(*t*) = *k*∙*t* + *c*, where *k* represents the slope of the change in fluorescence and is defined as *f∙(α-C*_*in*_*(t*_*0*_*)∙P*_*γ*_*∙n∙P*_*o*_*)/Vol*_*in*_ (see Materials and methods). Thus, the slope, *k*, is proportional and negatively correlated with permeability through a single hemichannel, *P*_*γ*_. Statistical comparisons of the estimated values of parameter *k* for each hemichannel type are shown in Fig. 3 G. The group comparison test showed significant differences in the medians (P val. <0.001). Using pairwise comparisons, *k* values did not differ significantly between Cx26 and Cx30-E49A hemichannels or between Cx30 and Cx26-A49E hemichannels. These data indicate that Cx26 and Cx30 hemichannels, like their GJ channel counterparts, differ in their anionic dye permeability characteristics and that the Ala/Glu difference at position 49 plays an important role in determining this differential permeability profile.

## Discussion

A number of studies have corroborated the differing charge permeability characteristics of Cx26 and Cx30 GJs. Permeabilities, largely determined from tracer flux studies, are in general agreement and indicate that Cx26 GJs, but not Cx30 GJs, are readily permissive to larger anionic molecules (Beltramello et al., 2003; Jagger and Forge, 2006; Manthey et al., 2001; Sun et al., 2005; Yum et al., 2007; Zhao, 2005). These characteristics of Cx26 and Cx30 GJs include studies both in exogenous expression systems and in cochlear supporting cells.

### Position 49 influences the passage of negatively charged dyes in Cx26 and Cx30 GJ channels

Using LY in a quantitative intercellular dye transfer assay, our data are consistent with previous findings regarding the anionic permeabilities of Cx26 and Cx30 GJs. We did observe LY transfer between Cx30-expressing cell pairs, rather than exclusion as suggested in some studies, but when junctional conductance as a relative measure of the number of conducting channels was taken into account, Cx30 was found to be considerably less permeable than Cx26. Extending these studies to the Ala/Glu variants, we found that swapping the residues at position 49, (A49E in Cx26 and E49A in Cx30) reversed this LY permeability profile, with Cx30(E49A) GJs showing a higher LY permeability compared with Cx26(A49E) GJs.

A reversal in the permeabilities to LY was reported in early exogenous expression studies in which both the cytoplasmic loop (CL) and C-terminal (CT) domains of Cx26 and Cx30 were exchanged (Manthey and Willecke, 2001). The construction of these chimeras was motivated by the fact that the CL and CT domains were the most divergent regions in these two otherwise closely related Cxs. This study, however, did not strictly assess permeability, but rather compared the number of neighboring cells showing fluorescence following microinjection of a single cell within a cluster or monolayer. Nonetheless, the results showed a substantive alteration in the characteristics of tracer spread. Given what is now known about the structures of Cx channels and the domains contributing to the pore, it seems likely that the exchange of the cytoplasmic domains may have produced structural changes that resulted in perturbations within pore-forming domains. The altered unitary conductances reported for these chimeras suggest that such alterations may have taken place.

Molecular dynamics simulations of Cx30, based on homology modeling using the Cx26 crystal structure, pointed to the first extracellular loop (E1) as a likely major structural determinant for the differing charge selectivities of these Cxs (Zonta et al., 2012). More precisely, the authors identified a positively charged lysine at the 41st residue (K41) in Cx26 and a negatively charged glutamate at the 49th position (E49) of Cx30 as main contributors to their differing electrostatic pore profiles and ionic permeabilities. Our data provides experimental support for residue 49 as an important contributor to these differences in permeability properties.

### The influence of Ala/Glu difference in defining anionic permeability extends to Cx26 and Cx30 hemichannels

Given that hemichannel docking, which is mediated by the extracellular loop domains, could affect the positioning of the pore-lining residues within E1, GJ and hemichannel configurations conceivably could differ in their permeability characteristics. Assessing the anionic tracer permeabilities of Cx26 and Cx30 hemichannels using a calcein efflux assay, we found that the anionic profiles are consistent between GJ channels and hemichannels. Cx26 hemichannels are readily permeable to calcein, whereas Cx30 hemichannels are virtually impermeable. Swapping the residues at position 49 strongly impacted this permeability difference in a similar manner as in the corresponding GJs, with Cx30(E49) hemichannels now showing robust calcein efflux and Cx26(A49E) showing reduced efflux. In previous studies using calcein to assess hemichannel and GJ function in cochlear supporting cells, no discernable effects of loading cells with calcein-AM were noted on Ca^2+^ wave propagation, which is mediated by Cx channels (Anselmi et al., 2008; Schütz et al., 2010).

Although permeability profiles of Cx26 and Cx30 are preserved between GJ channel and hemichannel configurations, the A49E substitution in Cx26 was found to produce a positive shift in the G-V relations of hemichannels, but not GJ channels (Sanchez et al., 2024). Computational studies suggest that conformational changes that mediate voltage-dependent closure of Cx26 hemichannels upon hyperpolarization are driven by rearrangements within a large network of interacting residues, influenced by those that reside in the pore and sense the electrical field (Kwon et al., 2012). Our results indicate that although the resulting changes in the relative conformational energies of open and closed states of Cx26 channels can differ in hemichannel and GJ channel configurations with an A49E substitution, the positioning of this residue in the pore is retained in both channel configurations along with its dominating influence on the passage of anions.

Our findings that permeability characteristics of Cx26 and Cx30 GJ channels and hemichannels are preserved is in agreement with a study of isolated cochlear supporting cells showing that Cx26 expression correlates with anionic tracer permeability, both in GJ channels and hemichannels (Zhao, 2005). However, a recent study examining anion fluxes using Alexa dyes in *Xenopus* oocytes expressing Cx26 or Cx30 reported that both hemichannels were permeable to negatively charged tracers with no difference between Cx26 and Cx30 (Xu and Nicholson, 2023). Extending studies to a physiologically relevant anionic signaling molecule, ATP, similarly showed ATP to be permeable and with no difference between Cx26 and Cx30 hemichannels. Cx26 GJs, however, exhibited an approximately sixfold higher ATP permeability compared with Cx26 hemichannels, and when comparing GJs, Cx26 GJs showed an approximately fourfold higher ATP permeability compared with Cx30 GJs. These findings suggest that the anionic permeability profiles previously ascribed to Cx26 and Cx30 GJs do not extend to hemichannels. Although ATP measurements accounted for conductance, dye fluxes did not, precluding a true assessment of their permeability differences.

Computational studies suggest that interactions between specific permeant molecules and the channel pore may contribute significantly to the energetics of permeation (Luo et al., 2016). Thus, regardless of whether GJ channels and hemichannels retain similar charge profiles, a given organic anion, such as ATP, may deviate from general expectations based on size and charge when compared with other chemically unrelated anions. Notably, however, the considerably reduced preference of Cx30 GJ channels for larger anions is consistent among several different tracer molecules.

Overall, we cannot exclude the possibility for some Cxs, GJ channel and hemichannel configurations differ in their permeability characteristics. This distinction could be Cx- and or permeant-specific. A cryo-EM structure of a hemichannel composed of Cx31.3 shows substantive differences compared with docked Cx26 and Cx46/50 hemichannels, notably in the positioning of the N-terminal domain at the cytoplasmic end of the pore (Lee et al., 2020). However, Cx31.3 and its rodent ortholog Cx29 do not form GJ channels (Ahn et al., 2008; Sargiannidou et al., 2008). Thus, there is no structural data on differences in electrostatic pore profiles for the same Cx in docked and undocked configurations. For a number of Cxs where there is electrophysiological data for both GJ channels and hemichannels, differences in functionality and susceptibility to regulatory agents, such as divalent cations, are established distinctions. However, regarding properties closely tied to pore-lining residues, GJ channels generally exhibit unitary conductances that are in accordance with the series addition of the hemichannels and voltage gating characteristics are generally correlative in both configurations (Bukauskas et al., 2006; Contreras et al., 2003; Harris, 2018; Srinivas et al., 2005; Trexler et al., 2000; Valiunas and Weingart, 2000; Verselis et al., 2000). We view that these data together with the corelative permeability effects of the Ala/Glu difference we observed in Cx26 and Cx30 GJ channels and hemichannels support a retained overall permeability profile in both channel configurations.

### Homomeric versus heteromeric channels

With both Cx26 and Cx30 extensively coexpressed in the cochlea, it is possible that most Cx channels exist as heteromers, suggesting that the permeability properties assessed from the corresponding homotypic channels may not reflect properties directly relevant to the native tissue. Given that the charge at position 49 in Cx30 robustly influences the passage of negatively charged molecules, heteromeric channels would likely show intermediate permeabilities, although the characteristics would depend on subunit stoichiometry and positioning.

Coexpression of Cx26 and Cx30 in HEK-293 cells were reported to mediate faster intercellular Ca^2+^ signaling than either of the respective homomeric GJ channel configurations (Sun et al., 2005). However, these studies did not take into account junctional conductance, which may be larger when both Cxs are expressed. In rodents, despite widespread co-expression in the organ of Corti and the lateral wall, Cx30 and Cx26 expression is not completely overlapping and show changes throughout development, indicating both homomeric and heteromeric channel assemblies, are likely to occur and that the distribution of channel types shows temporal variability (Ahmad et al., 2003; Forge et al., 2003b; Jagger and Forge, 2006). Moreover, super-resolution imaging of human cochlear tissue extracted from surgical procedures showed a preponderance of Cx26 and Cx30 arranged in separate clusters indicative that homomeric assemblies are the dominant form (Liu et al., 2016, 2017). Thus, despite the widespread expression of Cx26 and Cx30 in the cochlea, the properties of homotypic Cx26 and Cx30 GJs are relevant.

### Implications for cochlear function

The extensive network of GJ channels in the Organ of Corti serves, in part, to supply nutrients and signaling molecules that are needed for proper cochlear development (Chang et al., 2008; Zhang et al., 2005). Notably, a mechanism for cochlear pathogenesis for the inherited deafness mutation Cx26 (V84L) was ascribed to selectively impaired permeability to inositol 1,4,5-trisphosphate (IP_3_), which led to impaired Ca^2+^ wave propagation without changes in electrical coupling (Beltramello et al., 2005). Hemichannel permeability can also serve a critical role by mediating the uptake or release of factors that aid in cochlear development and function (Verselis, 2019). With differences in permeability characteristics, particularly those that could affect larger, negatively charged molecules such as IP_3_, cyclic-AMP, and ATP, it is conceivable that homomeric and heteromeric Cx26 and Cx30 GJ channels and hemichannels differ in the nature of the signals that are transmitted, which can affect cochlear function differently and/or at different times.

KO mouse models have shown that Cx26 can compensate for the loss of Cx30 in the acquisition of normal hearing, but conversely, Cx30 cannot compensate for the loss of Cx26 (Ahmad et al., 2007; Boulay et al., 2013; Qu et al., 2012). Differences in the developmental timing of Cx26 and Cx30 expression were suggested as a potential explanation, with the onset of Cx26 expression in the postnatal mouse cochlea preceding Cx30, thereby creating a time window during which Cx26 is indispensable (Qu et al., 2012). Increased Cx30 expression in the Cx26 KO mouse was achieved by transgenic integration of a bacterial artificial chromosome, which presumably contained the regulatory elements needed for proper spatial and temporal expression of Cx30. However, this scenario would still leave open the question of whether the replacement of Cx26 with Cx30 early on would preserve hearing. Also, cochlear development and the acquisition of hearing differs in humans so that similar timing arguments may not be valid for human hearing loss. Thus, in the absence of Cx26, the permeability difference exhibited by Cx30 GJ channels and hemichannels, as well as the reduced ability of Cx30 to function as a hemichannel described in our companion paper (Sanchez et al., 2024) could limit the intercellular and transmembrane transmission of signaling molecules vital for early cochlear development. Given that hearing loss is averted in Cx30 KO mice that preserve sufficient levels of Cx26 expression, it appears that homomeric Cx30 channels as well as heteromeric Cx26/Cx30 channels are dispensable for the acquisition of hearing and that homomeric Cx26 channels alone are sufficient. In this view, it is possible that the Cx30(E49A) variant of Cx30, which exhibits permeability characteristics more similar to Cx26, could circumvent these limitations, thereby preserving hearing in the absence of Cx26 both in rodents and humans. Notwithstanding, homomeric Cx30 channels, as well as heteromeric Cx26/Cx30 channels, can play sufficiently important roles in maintaining normal hearing over the long term as suggested by effects on hearing with deletion of Cx30 even in the presence of compensated levels of Cx26 (Forge et al., 2017).

## Supplementary Material

Table S1 shows statistical analysis for evaluation of Lucifer Yellow permeability through gap junction channels.

Table S2 shows the statistical analysis for the estimation of the number of functional hemichannels.

Table S3 shows the statistical analysis for the evaluation of calcein permeability through hemichannels.

## Data Availability

Original data from the article and supplementary material are available from the corresponding author upon reasonable request.
