# Supplementary material for: An Ala/Glu difference in E1 of Cx26 and Cx30 contributes to their differential anionic permeabilities
Source: J Gen Physiol. 2024 Sep 20;156(11):e202413600. doi: 10.1085/jgp.202413600 (PMC11415307; doi:10.1085/jgp.202413600)
Supplement: Table S1 — shows statistical analysis for evaluation of Lucifer Yellow permeability through gap junction channels. [file JGP_202413600_TableS1.docx]

**Table S1. Statistical analysis for evaluation of Lucifer Yellow permeability through gap junction channels.** Shapiro-Wilk normality test for the residuals of one-way ANOVA: W = 0.73596 (p-value = 2.311e-05). Group comparison of permeability coefficients using Kruskal-Wallis rank sum test: chi-squared = 18.048, df = 3, p-value = 0.0004299. Post hoc Dunn’s test for pairwise comparison of permeability coefficients:

|  | **Cx26** | **Cx26(A49E)** | **Cx30** |
| --- | --- | --- | --- |
| **Cx26(A49E)** | 3.689512  (p-value = 0.0001)* |  |  |
| **Cx30** | 2.770672  (p-value = 0.0028)* | -0.563544  (p-value = 0.2865) |  |
| **Cx30(E49A)** | 0.434965  (p-value = 0.3318) | -2.837612  (p-value = 0.0176)* | -2.105377  (p-value = 0.0176)* |

*denotes statistical significance.
