# Supplementary material for: An Ala/Glu difference in E1 of Cx26 and Cx30 contributes to their differential anionic permeabilities
Source: J Gen Physiol. 2024 Sep 20;156(11):e202413600. doi: 10.1085/jgp.202413600 (PMC11415307; doi:10.1085/jgp.202413600)
Supplement: Table S2 — shows the statistical analysis for the estimation of the number of functional hemichannels. [file JGP_202413600_TableS2.docx]

**Table S2. Statistical analysis for estimation of number of functional hemichannels.** Shapiro-Wilk normality test for the residuals of one-way ANOVA: W = 0.93543 (p-value = 0.1044). Group comparison of number of functioning hemichannels using one-way ANOVA: df = 3, SumSq = 123, MeanSq = 40.87, F value = 0.246 (p-value = 0.863). Post hoc Tukey‘s test for pairwise comparisons:

|  | **diff** | **lwr** | **upr** | **p adj** |
| --- | --- | --- | --- | --- |
| **Cx26(A49E)-Cx26** | **5.1831125** | **-15.23253** | **25.59875** | **0.8940286** |
| **Cx30-Cx26** | **4.3446792** | **-14.99569** | **23.68505** | **0.9233318** |
| **Cx30(E49A)-Cx26** | **4.5728125** | **-13.96135** | **23.10698** | **0.9016592** |
| **Cx30-Cx26(A49E)** | **-0.8384333** | **-22.52332** | **20.84645** | **0.9995428** |
| **Cx30(E49A)-Cx26(A49E)** | **-0.6103000** | **-21.57931** | **20.35871** | **0.9998046** |
| **Cx30(E49A)-Cx30** | **0.2281333** | **-19.69549** | **20.15176** | **0.9999881** |
