# Supplementary material for: An Ala/Glu difference in E1 of Cx26 and Cx30 contributes to their differential anionic permeabilities
Source: J Gen Physiol. 2024 Sep 20;156(11):e202413600. doi: 10.1085/jgp.202413600 (PMC11415307; doi:10.1085/jgp.202413600)
Supplement: Table S3 — shows the statistical analysis for the evaluation of calcein permeability through hemichannels. [file JGP_202413600_TableS3.docx]

**Table S3. Statistical analysis for evaluation of calcein permeability through hemichannels.** Shapiro-Wilk normality test for the residuals of one-way ANOVA: W = 0.96896 (p- value = 8.295e-05). Group comparison of permeability coefficients using Kruskal-Wallis rank sum test: chi-squared = 22.15, df = 3, p-value = 6.071e-05. Post hoc Dunn’s test for pairwise comparison of permeability coefficients:

|  | **Cx26** | **Cx26(A49E)** | **Cx30** |
| --- | --- | --- | --- |
| **Cx26(A49E)** | -2.986333  (p-value = 0.0014)* |  |  |
| **Cx30** | -3.705530  (p-value = 0.0001)* | -1.194031  (p-value = 0.1162) |  |
| **Cx30(E49A)** | -1.259191  (p-value = 0.1040) | 2.623804  (p-value = 0.0043)* | 3.636702  (p-value = 0.0001)* |

*denotes statistical significance.
